# Fabrication Techniques for Graphene Oxide-Based Molecular Separation Membranes: Towards Industrial Application

**DOI:** 10.3390/nano11030757

**Published:** 2021-03-17

**Authors:** Ohchan Kwon, Yunkyu Choi, Eunji Choi, Minsu Kim, Yun Chul Woo, Dae Woo Kim

**Affiliations:** 1 Department of Chemical and Biomolecular Engineering, Yonsei University, Yonsei-ro 50, Seodaemun-gu, Seoul 03722, Korea; titan@yonsei.ac.kr (O.K.); ykgs26@yonsei.ac.kr (Y.C.); v2choiej@yonsei.ac.kr (E.C.); yondu1117@yonsei.ac.kr (M.K.); 2 Department of Land, Water and Environment Research, Korea Institute of Civil Engineering and Building Technology (KICT), Goyang-si, Gyeonggi-do 10233, Korea; yunchul84@kict.re.kr; 3 Department of Construction and Environment Engineering, University of Science and Technology (UST), Daejeon 34113, Korea

**Keywords:** graphene oxide, fabrication methods, scale-up, separation, membrane

## Abstract

Graphene oxide (GO) has been a prized material for fabricating separation membranes due to its immense potential and unique chemistry. Despite the academic focus on GO, the adoption of GO membranes in industry remains elusive. One of the challenges at hand for commercializing GO membranes lies with large-scale production techniques. Fortunately, emerging studies have acknowledged this issue, where many have aimed to deliver insights into scalable approaches showing potential to be employed in the commercial domain. The current review highlights eight physical methods for GO membrane fabrication. Based on batch-unit or continuous fabrication, we have further classified the techniques into five small-scale (vacuum filtration, pressure-assisted filtration, spin coating, dip coating, drop-casting) and three large-scale (spray coating, bar/doctor blade coating, slot die coating) approaches. The continuous nature of the large-scale approach implies that the GO membranes prepared by this method are less restricted by the equipment’s dimensions but rather the availability of the material, whereas membranes yielded by small-scale methods are predominately limited by the size of the fabrication device. The current review aims to serve as an initial reference to provide a technical overview of preparing GO membranes. We further aim to shift the focus of the audience towards scalable processes and their prospect, which will facilitate the commercialization of GO membranes.

## 1. Introduction

Membrane separation has gained significant attention due to its distinctive advantages over distillation and adsorption-based processes [1,2,3]. Facilitated by potential differences, the technique allows continuous, energy-efficient operations compared to traditional separation processes. Furthermore, the modular characteristics of the technology enable easy scalability with minimal managing costs. For membrane separation, the most crucial aspect lies in the designing of the selective medium. Due to such reasons, studies focused on developing and optimizing novel materials with high selectivity and flux have been commissioned. Out of various candidates, graphene oxide (GO) derivatives are a popular choice among researchers. Unique properties such as high mechanical, chemical, and thermal stability work in favor of the material’s wide acceptance. However, above all, the most appealing feature comes from its morphology. GO consists of an atomic monolayer carbon sheet with attached oxygen groups, making them a stereotypical 2D material. This high aspect ratio structure allows facile stacking of layers where numerous approaches have been pursued to yield different membranes with varying functionality [4,5,6].

As of now, most studies have been focused on modifying GO’s intrinsic chemistry [7,8,9]. Transport phenomena in a typical GO-based membrane occur through the interlayer spacing of the flakes, where separation properties arise due to the variances in the molecule’s ability to pass through these channels [10,11,12]. As a result, many of the reported works focus on tuning the interlayer distances to separate specific molecules [8,13,14]. Generally, for gas separation, low-defect membranes with interlayer spacing below 0.7 nm are frequently preferred, whereas, for water nanofiltration, GO membranes with interlayer spacing up to 1.0 nm are frequently reported at the solvent-swelling state and the interlayer spacing can be further expanded depending on the type of solvents. Well-structured GO structures have uniform distances that allow selective and facile mass transport. Thus, at the academic level, circumstances encourage small-scale, batch-unit approaches as these methods provide better controllability of the experiment. Furthermore, limiting the membrane area also benefits by reducing structural defects, minimizing efforts in quality control.

On the other hand, to satisfy commercial demands, large-scale, continuous manufacturing processes are highly desired. This apparent disparity of reported and required preparation processes captures the barrier between GO membranes and their industrial acceptance. Fortunately, there has been a growing consensus on the topic within the field [15,16,17]. As such, recent efforts fueled several investigations utilizing bar-coating, spray coating, and slot-die coating, which are known scalable processes.

The current review recognizes this rising movement and aims to provide much-needed clarification regarding the advances in fabrication pathways for GO-based separation membranes. Although lab-scale production methods will be discussed as a reference, note that the reader’s focus should be gently guided to scalable techniques and their potentials. The scope of the discussion is also limited only to the physical fabrication methodologies because many works of literature already discussed the chemistry of GO and its mechanism for membrane applications.

## 2. Overview

**Figure 1:**
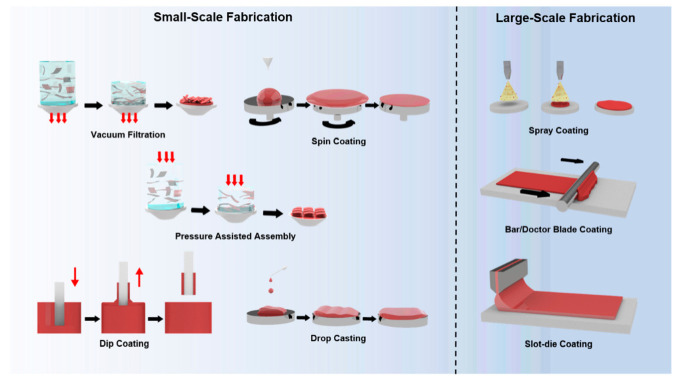
Widely adopted graphene oxide (GO) membrane fabrication methods; classified by scalability.

In this review, a total of eight different methodologies are examined (Figure 1). These strategies have been widely adopted by numerous studies to manufacture GO derivative membranes for gas separation, nanofiltration, pervaporation, gas-barrier application, and desalination. Although GO films are further employed for various uses, namely as electrodes, we have narrowed the search to separation technologies exclusively. The discussion at hand identifies each method’s pros and cons to provide insights into the facile synthesis of GO membranes. Among the eight methodologies, five are considered small-scale techniques, namely vacuum filtration, spin coating, pressure-assisted assembly, dip coating, drop-casting. The inherent property of these small-scale methods is that they are operated on a batch basis. Meanwhile, the remaining three, spray coating, bar/doctor blade coating, and slot-die coating, are addressed as large-scale techniques, as these pathways can produce membrane continuously and the fabrication area is only limited by the feature dimension of the equipment.

## 3. Small-Scale Fabrication Techniques

Small-scale methods share a common property of batch unit production. Commonly, the following five methods are applied in lab settings as they can fabricate thin graphene layers with a well-defined morphology more easily. Even if scale-up using the small methods is challenging, the small-area membrane can be good enough to understand the effects of a graphene structure on membrane performance. Therefore, most of the published works employ small-scale methods to fabricate GO membranes.

### 3.1. Vacuum Filtration

Out of all the methods, vacuum filtration is the most popular method for GO membrane production. A diluted GO dispersion is filtered through a porous substrate where a vacuum is forced at the other side of the filter. The pressure gradient directs the solvent flow through the substrate which deposits the dispersed GO flakes, constructing a selective layer. By varying the concentration, the thickness can be readily controlled from few nanometers to micrometers, where thicker films can be delaminated from the substrate to fabricate freestanding structures [18]. Despite its simplicity, the process consumes large amounts of solvents and time to fabricate membranes. Moreover, the size of the products is mostly limited in the few centimeter scales. Thickness is another factor to consider, as thicker membranes may have a different alignment depending on their depth. Since the pressure is stronger closer to the substrate, thicker membranes may have a more disordered morphology at the top due to the weaker guiding force [19]. Moreover, the longer filtration time required for preparing the thick GO assemblies hampers facile production. In addition, the thicker GO film is beneficial for gas barrier coating rather than membrane applications.

Yang et al. fabricated 10 nm-thick GO membranes on porous alumina and nylon porous support by using vacuum filtration and the membrane was employed for organic solvent nanofiltration (OSN) [20]. While relatively large GO flakes with a diameter of 10~20 µm were deposited, fast organic solvent permeance (1~10 LMH/bar depending on the viscosity of solvents) was achieved because of the ultrathin thickness of the membrane and the presence of pinholes between the edges of the stacked GO layers. The membrane was selective for nanoscale dye molecules in methanol, showing rejection above 95% in the dead-end filtration. Huang et al. also reported OSN membranes fabricated by the vacuum filtration method [21]. The study employed reduced GO (rGO) flakes accumulated on porous alumina or nylon filters, which were solvated with organic solvents before being completely dried to avoid irreversible packing of the layers. The thickness of the studied membranes was 18 to 25 nm. As the interlayer spacing was expanded by the solvation, organic solvent permeance was significantly enhanced than that of the membrane without solvation (up to 80 LMH/bar for methanol). The solvation method may be applicable only on the lab-scale, implying that controlling the interlayer spacing is critical to enhancing the permeance of organic solvents through stacked graphene layers.

**Figure 2:**
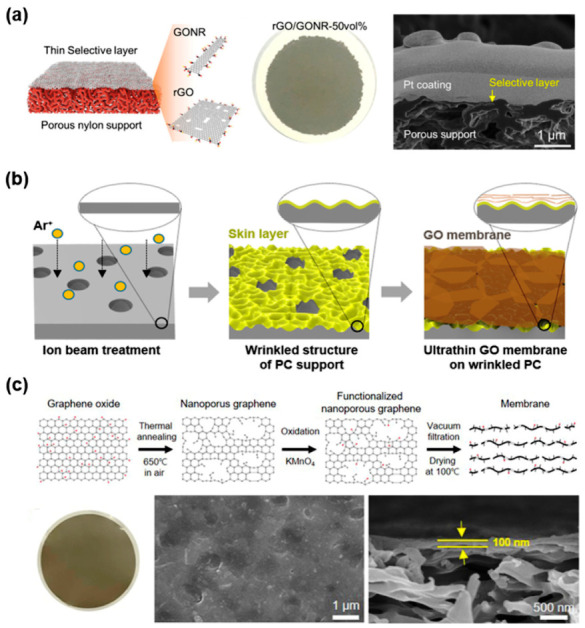
(**a**) GO nanoribbon (GONR)/GO composite membrane prepared by vacuum filtration [22]. Copyright 2019 American Chemistry Society; (**b**) GO layer deposition on wrinkled substrates for high water flux [23]. Copyright 2019 Elsevier; (**c**) nanoporous GO sheet membranes for nanofiltration application [24]. Copyright 2021 Elsevier.

The vacuum filtration technique has also been widely utilized for fabricating hybrid and structured graphene membranes for dye and ion separation. Cho et al. significantly increased the water permeance of nanofiltration membranes to 312.8 LMH/bar by depositing a rGO and GO nanoribbon (GONR) mixture on a porous support (Figure 2a) [22]. The high flux was a result from the nanochannels being enlarged by the intercalated GONR strips inside the rGO layer. The dispersions were separately prepared and mixed before vacuum filtering through a porous support. Membrane thickness was reported as 30 nm. Nam et al. proposed a wrinkled substrate by ion beam etching to increase water permeance (Figure 2b) [23]. The uneven surface hindered the stacking of GO flakes and widened the interlayer distance from 0.89 to 0.92 nm. The wider peaks of the X-ray diffraction spectra also indicate a larger disorder in the alignment of the GO sheets. Furthermore, free volumes between the GO layer and the substrate also increased water transport. This suggests that, although vacuum filtration can be exploited to readily stack various 2D materials, careful considerations must be made in selecting substrates, as the alignment of the sheets is affected by the morphology of the support. Kang et al. prepared nanoporous rGO sheets by a rapid thermal treatment (Figure 2c) [24]. The nanoporous rGO sheets were dispersed in water by a secondary functionalization process before depositing on substrates by vacuum filtration. The resulting membrane had a thickness of 100 nm. For the nanofiltration test, the evolved pores of the rGO sheets allowed the fast permeance of water (586 LMH/bar). Particularly for the graphene materials with small amounts of oxygen-functional groups, selecting the proper solvent is critical, as some organic solvents dissolve the polymer support. Commonly, dimethylformamide or N-methyl-2-pyrrolidone is used as a solvent for preparing graphene dispersion, which can dissolve most of the polymer support. Otherwise, graphene is required to be functionalized to be soluble in water or eco-friendly solvents.

### 3.2. Pressure-Assisted Assembly

**Figure 3:**
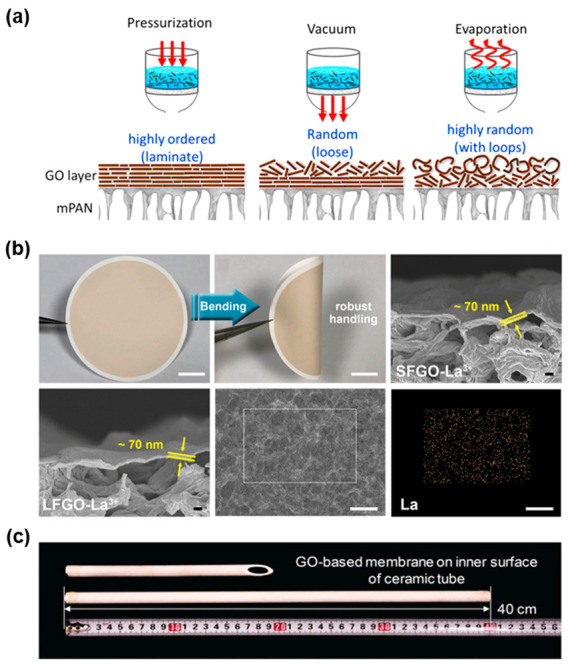
(**a**) Comparative analysis of various filtration-based methods for GO membrane preparation [19]. Copyright 2015 Elsevier; (**b**) La^3+^ ion crosslinked small flake GO membranes for nanofiltration [14]. Copyright 2020 The American Association for the Advancement of Science; (**c**) high-stability GO layers by molecular bridging on ceramic tube type membranes [13]. Copyright 2020 John Wiley and Sons.

Similar to the vacuum filtration method, pressure-assisted assembly incorporates a filtration process to produce selective membranes. Instead of negative pressure at the opposite of the GO layer, positive pressure is applied with the GO dispersion to deposit the carbon layer on substrates. Tsou et al. performed a comparative investigation of membranes yielded by both vacuum and pressure filtration (Figure 3a) [19]. The pressure-assisted method created a well-stacked, even morphology through the in-plane of the membrane, whereas the vacuum filtration method had a relatively loose structure with less ordered layers at the top. Moreover, the pressurized approach produced significant thickness reduction from 384 to 231 nm due to the increased ordering. These membranes were utilized in pervaporation experiments. Zhang et al. reported ion sieving membranes by coating a polyelectrolyte layer on GO membranes by using the pressure-assisted technique [25]. The membrane’s overall width was 15 cm in diameter, and the thickness was around 100 nm. The surface charge of the polyelectrolyte layer successfully controlled the ion selectivity of the membrane. Hung et al. also reported GO membranes prepared by pressure-assisted filtration for isopropanol-water pervaporation [26]. The cross-sectional analysis confirmed the highly ordered state of GO sheets with varying thicknesses from a few nanometers to 1 micrometer, indicating that the pressure-assisted method is effective to prepare a well-ordered thick GO layer.

Yuan et al. fabricated nanofiltration membranes with COOH functionalized GO flakes [27]. The morphology exhibited higher surface wrinkles, which were evolved during the filtration process. These anomalies were formed during the draining process, where the functionalized GO sheets increased water affinity, shaping larger amounts of drain sites. Nie et al. utilized small GO flakes, which were deposited on nylon substrate to prepare OSN membranes (Figure 3b) [14]. These GO membranes were further crosslinked with cations such as La^3+^ to increase stability. Due to the GO sheets’ small lateral dimension, less tortuous pathways were formed, which increased the transport through the membranes. Pressure-assisted filtration method can also be employed for applying GO layers on hollow fiber. Recently, Zhang et al. successfully coated the inner surface of ceramic tubes with GO laminates (Figure 3c) [13]. In this study, an interfacial long-chained molecular bridge anchors the sheets to the ceramic substrate while intercalated molecules between the flakes limit the membrane’s swelling. The bridges’ overall synergy increased the stability, which was confirmed by the 600 h cross-flow operation time.

### 3.3. Spin Coating

**Figure 4:**
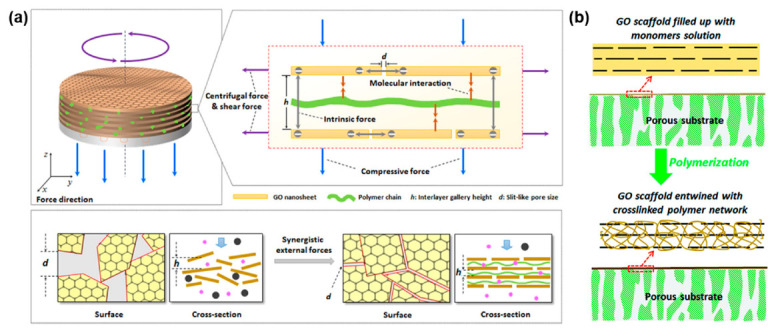
(**a**) Vacuum-assisted spin coating technique for highly aligned gas separation GO membranes [30]. Copyright 2016 American Chemical Society; (**b**) crosslinked polymer GO membranes for OSN application [31]. Copyright 2018 American Chemical Society.

Spin coating is another method widely explored in lab settings. During the technique, a substrate is placed on a rotating disc, on which the GO dispersion is applied in a dropwise fashion. The method is beneficial to form a well-aligned GO layer due to the centrifugal shear force and the ultrathin layer also can be fabricated. Kim et al. demonstrated that the spin-coated few-layer graphene sheet membranes with a thickness from 3 to 10 nm can be effective for H_2_/CO_2_ separation [28]. Chi et al. reported 20 nm-thick GO membranes prepared by spin coating large GO flakes on porous alumina substrate and the large GO with a diameter of 20 μm was synthesized by a mild freeze–thaw approach [29]. The fabricated membrane showed gas separation performance with H_2_ permeance of 3.5 × 10^−7^ mol/(m^2^·s·Pa) and H_2_/CO_2_ separation factor of 240. Shen et al. produced GO membranes by a vacuum-assisted spin method (Figure 4a) [30] and proved that the additional vacuum force further compacts the interlayer spacing, resulting in the nanochannel with 0.4 nm height. The report further compared other techniques such as drop, dip-casting, filtration, and spin coating methods, and concluded that the vacuum-spin approach produces membranes with slightly lower flux at around 1000 barrer but higher H_2_/CO_2_ selectivity at 30.

The spin coating has also been used for realizing liquid-based filtration membranes. Kim et al. spin-coated a mixture of GO-monomer dispersion to fabricate chlorine tolerant membranes for forward osmosis (Figure 4b) [31]. The composite membrane’s thickness was measured as 26.3 nm and showed a salt rejection of 99.9% with a water flux of 25.8 LMH. Likewise, Dong et al. reported increased ion rectification after spin coating a GO layer on conical nanopore membranes [32]. The polymer support was first irradicated and etched to create conical-shaped pores. The GO layer acted as a cation absorber and source to increase the ion selectivity. In general, spin-coating is an effective method for preparing high-quality GO membranes with well-oriented and stacked morphology. Whereas the produced membranes typically have the smallest lateral dimensions due to the size limitation of the processing equipment, the diameter of the substrate is usually smaller than 8 inches. Most of all, the method is applicable for substrates with a smooth surface. Otherwise, a defective structure can be formed during the spinning process.

### 3.4. Dip Coating

**Figure 5:**
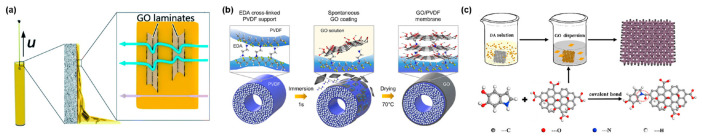
(**a**) Alignment of GO sheets by shear force during the pulling process [33]. Copyright 2017 Royal Society of Chemistry; (**b**) GO-coated hollow fiber membranes enabled by ethylenediamine functional groups for nanofiltration [34]. Copyright 2020 American Chemical Society; (**c**) GO-coated metal mesh substrates for oil-water separation [35]. Copyright 2019 Elsevier.

The process of dip-coating is by far one of the most intuitive approaches. The technique requires a stock GO dispersion in which the substrate is lowered to soak. As the support is pulled from the bulk dispersion, the GO layer is dried, which leaves behind a coating. The thickness of the layer can be controlled by various factors such as temperature, concentration, and the substrate’s removal speed. The technique’s facileness does come at a cost regarding the membrane’s quality, where it is often harder to prepare uniform GO sheet structures. Nonetheless, with rigorous optimization, several studies were able to synthesize well-aligned membranes successfully. Zhang et al. dip-coated hollow fiber Pebax membranes, which increased the N_2_/CO_2_ selectivity (Figure 5a) [33]. The substrate’s pulling speed was controlled to yield aligned GO sheets on the surface where the optimized rate was 0.4 cm/s.

Goh et al. synthesized polyamide-imide hollow fiber membranes by a dry-jet wet spinning process [36]. After this, the surface of the membranes was positively charged by attaching polyethyleneimine branches. Due to the positive charge, when the fiber was soaked in a GO dispersion, negatively charged GO sheets adhere instantly to the exterior, creating a selective layer. Similarly, Eum et al. fabricated polyvinylidene fluoride hollow fiber membranes with ethylenediamine functionalization (Figure 5b) [34]. GO layer was coated to the fibers by a simple immersion experiment where the ethylenediamine groups and the sheets were covalently bonded. The resulting membrane had a molecular cutoff of 0.8 nm with a rejection of 95%. Shen et al. also dip-coated polydopamine-functionalized tubular ceramic membrane for increasing the nanofiltration performance [37]. Solvent green was mixed into the GO dispersion for the experiment, which ultimately enlarges the interlayer spacing between the layers from 0.77 to 0.89 nm. The aromatic rings of solvent green attach to individual GO flakes by the π–π interaction, which then intercalates between the layers to widen the spacing. As a result, a significant increase in water permeance was observed from 56.8 to 330 LMH/MPa.

Another advantage of dip-coating methods is that the process is relatively insensitive to the substrate’s shape. Yin et al. were able to fabricate a stainless-steel mesh decorated with a GO layer (Figure 5c) [35]. To increase the deposition of the GO layer, the mesh was first functionalized with a polydopamine coating. Both steps were performed by the dip-coating method. Later, the mesh was employed in a water-oil separation experiment where the increased hydrophilicity from the GO layer benefited the rejection of the oil phase.

### 3.5. Drop Casting

**Figure 6:**
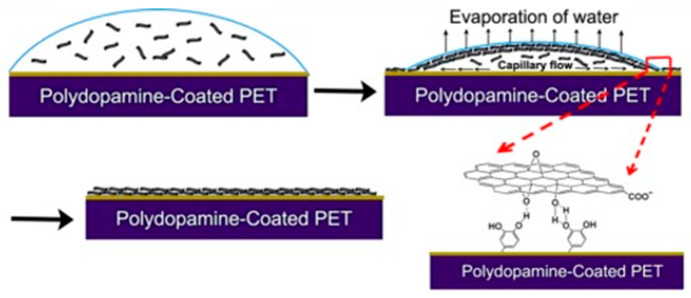
Mechanisms for rGO layer deposition for drop-casting method [38]. Copyright 2014 Elsevier.

Drop casting is another rudimentary approach often utilized which has similar properties to dip coating. The process involves dropping GO dispersions on substrates, which are then left to dry. The technique is arguably the most rudimentary form of fabricating GO layers. Given the lack of surface ordering force, it is difficult to align the GO sheets, decreasing the quality of the membrane. Nonetheless, there are several cases where drop-casting was used to produce separation membranes. Zhao et al. demonstrated rGO coatings on surface-modified polymer supports (Figure 6) [38]. The research employed polydopamine treated surfaces to adhere the rGO flakes and further discussed the detailed deposition mechanism during the drying process. These films were not explored for separation applications. Sun et al. prepared GO membranes by the drop-casting method, which were used for ion sieving application [39]. These freestanding membranes had a thickness under 10 μm with a rather coarse surface. The membrane was able to separate heavy metal ions, and organic contaminates while leaving sodium ions to permeate. Church et al. also fabricated self-standing GO membranes by drop-casting [40]. The GO dispersions were also mixed with various ions (NH_4_OH, NaOH, NH_4_Cl, NaCl, or CaCl_2_) which would later hinder ethanol movement during the pervaporation experiments.

Contrary to its simplistic manner, expanding membrane dimensions with drop-casting is a difficult task. Fabrication is dependent on solvent evaporation which hampers rapid processing. Furthermore, this aspect also makes quality control difficult with larger dimensions.

## 4. Large-Scale Fabrication Techniques

One of the issues within the literature is that there is no definite standard to classify what is and what is not scalable. It seems that the labeling is up to the author’s claim, where if the method has even the slightest potential to be expanded, the fabrication is termed as a scalable process. In a stricter sense, this can be misleading. We emphasize that to be truly considered scalable, the limiting factor for membrane dimensions should not be dependent on the size of the equipment as in batch unit production. Rather, the size of the yielded product should be proportional to the available substrate. Furthermore, often the most critical challenge of sizing up a process is not related to the actual sizing up of the equipment. Instead, the major issue is with the consistency of the product. Maintaining pristine quality level is inherently more difficult with batch unit production methods, which were addressed in the previous section. Thus, the current review suggests a harsher criterion that states that fabrication methods considered scalable are limited to those that continuously produce GO membranes. By the virtue of their properties, as long as a constant feeding of material is maintained, a membrane will be extruded as a final product. The three processes selected in this section can be coupled with a roll-to-roll setup to increase the production efficiency which may facilitate industrial adoption.

### 4.1. Spray Coating

**Figure 7:**
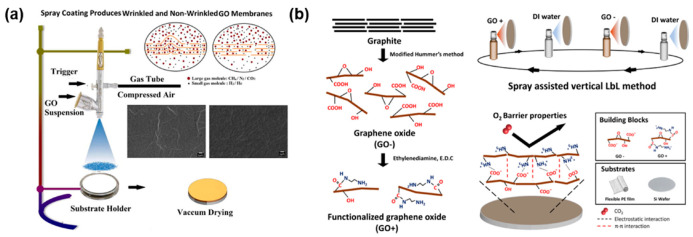
(**a**) Nonwrinkled GO membranes prepared by the spray-coating method [41]. Copyright 2018 Elsevier; (**b**) alternating charged GO sheet deposition for O_2_ barrier films [42]. Copyright 2019 Nature Publishing Group.

Spray coating utilizes a spray gun that forms the GO dispersion into minuscule droplets, which are then scattered on desired surfaces. GO layers are formed as the solvent dries from the substrate. Similar to the dip-coating technique, spray coating is relatively insensitive to the shape of the substrate. Although spray coating is undoubtedly a scalable process, there is a significant tradeoff relating to the membrane’s quality as the products may lack uniformity. Ibrahim et al. prepared gas separation membranes through spray-coating (Figure 7a) [41]. The study also reported that, compared to filtration methods, spray coating membranes have less wrinkle formation, which reduces the GO sheet’s interlayer spacing, thus increasing the experiment’s selectivity. Heo et al. fabricated oxygen barrier GO films by a layer-by-layer approach (Figure 7b) [42]. Two types of GO were prepared: negatively charged pristine GO and positively charged amine group functionalized GO. The two dispersions were alternatively spray-coated onto a substrate. The opposing charge of the GO layer increased the adhesion, resulting in a reduction in O_2_ permeance.

As previously mentioned, spray coating can be applied to substrates with various shapes. Mahalingam et al. successfully applied a GO layer on polyetherimide hollow fibers for nanofiltration [43]. The resulted membranes had a high water and acetone flux of 40 and 24 LMH/bar, respectively, and a Rose Bengal rejection of 90% in acetone.

### 4.2. Bar/Doctor Blade Coating

Bar coating and doctor blade coating is the most widely utilized technique for GO membrane fabrication among the scalable methods. Concentrated GO dispersions can acquire viscoelastic behavior due to the hydrogen bonding existing between the water molecules and GO sheets’ oxygen functional groups. During the coating procedure, a shear force can be induced to the viscous gels, enabling the alignment of the GO laminates. This method allows us to yield high-quality GO membranes with a thickness as low as 100 nm but becomes difficult to prepare in the 10 nm scale. Another disadvantage is that the method is sensitive to the shape of the substrate, where the target surface must have a leveled exterior.

**Figure 8:**
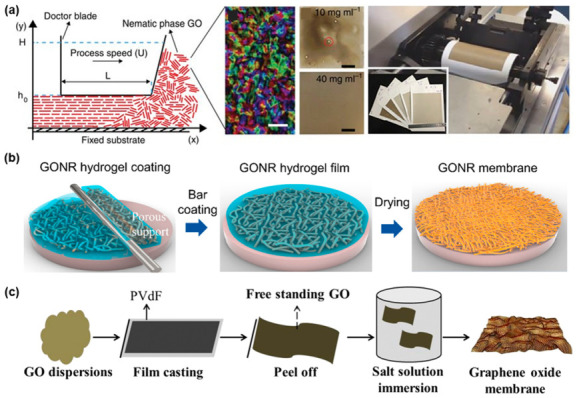
(**a**) Scalable production of GO membranes enabled by nematic phase GO alignment [16]. Copyright 2016 Nature Publishing Group; (**b**) GONR hydrogel membrane fabrication for nanofiltration [17]. Copyright 2020 American Chemical Society; (**c**) crosslinked, freestanding GO membranes for ion rejection [47]. Copyright 2019 Royal Society of Chemistry.

Akbari et al. demonstrated the scalable fabrication of GO membranes by employing a gravure printing machine (Figure 8a) [16]. In this research, the GO dispersion was first concentrated by adding dry hydrogel, which would soak the additional water. A doctor blade was used to coat the nylon substrates with nematic phase GO dispersion while simultaneously inducing shear force. The concentration of the dispersion used was above 40 mg/mL, which enabled highly ordered GO layers. The produced membranes were further tested for nanofiltration, where aqueous dye and ion rejection experiments were performed. The group in a separate study tested the membrane for OSN application and produced a new scale factor linking permeance to the solvent’s viscosity [44]. Choi et al. were also able to prepare GONR gels for membrane fabrication through bar coating (Figure 8b) [17]. The GONR gels exceeding 50 mg/mL concentration started to form scaffolds by self-assembly. The synthesized membranes had high water permeance of 8 LMH/bar and showed impressive stability for 48 h under 6 bar, cross-flow experiment conditions. Mahalingam et al. utilized protic ionic liquid (ethylammonium nitrate) to increase the viscosity of low concentration GO dispersion [45]. The study reports that the balance of electrostatic forces between GO sheets and the ionic liquid allows for better alignment. The ionic liquid phase was removed before testing nanofiltration performances by immersing in an acetone bath. Chang et al. prepared nanoporous rGO membranes by rod coating for ion and dye rejection experiments [46]. Pristine GO sheets were made porous by oxidizing with nitric acid. The GO layer membranes were later reduced under hydrogen in the presence of Pd catalyst. The research reported that elongation of the reduction time has an apparent tradeoff between flux and selectivity.

Bar coating can also be readily used to fabricate freestanding GO membranes. Ghaffer et al. produced GO membranes without substrate, which were later intercalated with various cations (K^+^, Ba^2+^, Na^+^, Ca^2+^) and further reduced to limit the interlayer distances (Figure 8c) [47]. These membranes were used for ion rejection tests and showed high retention of multivalent ions, namely Pb, Ni, Cu, and Mg.

### 4.3. Slot-Die Coating

**Figure 9:**
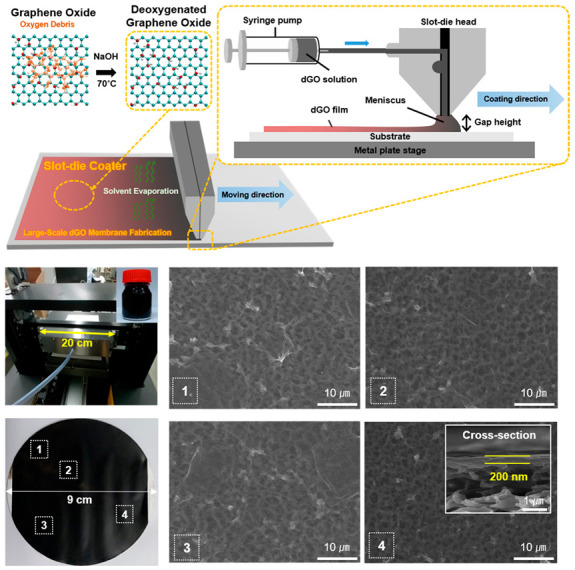
Scalable production of GO membranes by slot-die coater [15]. Copyright 2020 Elsevier.

Recently, Kim et al. demonstrated GO membrane fabrication by employing a slot die coater (Figure 9) [15]. The study reported that 100 nm-thick membranes with partially deoxygenated GO sheets were prepared through the slot-die coating method and showed satisfactory stability under aqueous conditions. The membranes’ performance was tested under cross-flow filtration, which marked a rejection rate of 99% for sub-nanometer dye molecules with a high water flux of 30 LMH/bar. Slot-die coaters are unique because well-aligned GO membranes can be realized by the shear force which occurs between the liquid meniscus and the moving substrate while avoiding some inherent issues with bar coating. Firstly, the technique allows one to utilize GO dispersions with relatively lower viscosity (watery GO solution with concentration below 10 mg/mL). This aspect permits fabricating thinner membranes without rigorous pretreatment of the GO stock solution. Secondly, the thin nature of the wet layer enables spontaneous drying, which reduces the required time for fabrication. Thirdly, as GO is directly coated on a target substrate, minimal waste of stock solution is produced. Lastly, the technique can be further explored to utilize different materials for membrane production.

## 5. Conclusions

The current survey highlights eight different methods for GO membrane fabrication and classifies them by their scalability. Although the focus here has been on GO membranes, these approaches can be generalized to realizing membranes with various 2D materials. The criterion for scalable production imposed in this review is that the membrane must be manufactured continuously. Small-scale approaches such as vacuum filtration method, drop-casting, dip coating act as a facile pathway for investigating GO membrane chemistry and transport mechanism. Spin coating and pressure-assisted assembly realizes well-ordering of the GO sheets. Conversely, large-scale methods allow for practical studies of GO membranes. Techniques such as slot-die and bar coating uniquely align the flakes with the inducement of shear force on planar supports, whereas spray coating is more suitable for irregular substrates.

GO has been widely utilized in separation processes because of its ability to form thin membranes with controllable selectivity and high permanence. There is much research regarding GO membranes and their potential in various application areas, such as water purification, gas separation, organic solvent filtration, and battery separator. Nonetheless, the challenge at hand should not just be focused on depicting its possibilities, and the center of discussion needs to gradually shift towards large-scale continuous membrane fabrication techniques and its module fabrication. Most of all, membrane evaluation is required in practical operating conditions such as cross-flow filtration with high concentration solvent at high pressure and fast feed flow. This review is an effort to provide insights into pathways that enable the transition.

## Data Availability

No new data were created or analyzed in this study. Data sharing is not applicable to this article.
